# Prevalence of dementia in Colombian populations

**DOI:** 10.1590/S1980-57642014DN84000004

**Published:** 2014

**Authors:** Efraín Amaya Vargas, Ángela Magnolia Ríos Gallardo, Guillermo González Manrrique, Lina M Murcia-Paredes, María Consuelo Angarita Riaño, Grupo DNEUROPSY

**Affiliations:** 1 Neurologist, Professor of Medicine Program and Member of the Group DNEUROPSY - Surcolombiana University; 2 Psychologist, PhD Psychology Focusing on Cognitive Neuroscience. Teacher and Director of the Group DNEUROPSY - Surcolombiana University; 3 Neurologist. Health Specialist in Management. Head of the Area of Neurophysiology University Hospital in Neiva. Professor and Member of the Group DNEUROPSY - Surcolombiana University; 4 Psychologist. Specialist in Health Psychology, Masters Student in Psychology. Member of Group DNEUROPSY - Surcolombiana University; 5 General Medical. Specialist in Management of Health Organizations. Master's Student in Public Health. Area Leader for Public Health. Department of Health - Huila; 6 Research Group in the Development of Neurosciences and Psychology - Surcolombiana University

**Keywords:** dementia - epidemiology, prevalence, caregivers, health-care services for older people - economy, health priorities, prevalence of dementia in Neiva and Colombia

## Abstract

With the gradual increase in the life expectancy of the population due to scientific progress and public health at the service of society, the prevalence of dementia has been increasing at different rates worldwide. Currently, the prevalence rates range between 5% and 7% (6.4% in the U.S. and up to 8.5 % in Latin America) in subjects older than 60 years. The lowest prevalence rate (2.1%) has been reported from sub-Saharan Africa, probably due to selective mortality under 60 years of age. By contrast, a very high prevalence of dementia (23.6% dementia in individuals >60 years) was observed in the city of Neiva, Southern Colombia. We believe that this high rate could be explained by the presence of several risk factors such as very low schooling, low socio-economic strata, chronic diseases, the inclusion of geriatric homes among others, and additional unknown factors.

## Abstract (pt)

Com o aumento gradual da expectativa de vida das pessoas, devido aos progressos científico e de saúde pública a serviço da sociedade, a prevalência de demência tem aumentado a taxas bastante divergentes em todo o mundo. Atualmente, as taxas de prevalência variam entre 5% e 7% (6,4% em os EUA até 8,5% na América Latina) em indivíduos com idade superior a 60 anos. Prevalência mais baixa (2,1%) foi relatada na África sub-saariana, provavelmente devido a mortalidade seletiva abaixo dos 60 anos de idade. Ao contrário, uma alta prevalência de demência (23,6% de demência em pessoas >60 anos) foi observada na cidade de Neiva, no sul da Colômbia. Acreditamos que essa taxa alta pode ser explicada pela presença de vários fatores de risco, como: baixa escolaridade, níveis socio-económicos mais baixos, doenças crônicas, inclusão de instituições geriátricas, entre outros, e outros possíveis fatores desconhecidos.

## INTRODUCTION

**History and definition of dementia**. The Colombian medical literature^1^ discusses the historical evolution of the dementia concept from 2000 BC to 2000 AC. The World Health Organization (WHO) defines dementia as a syndrome caused by a brain disease usually of a chronic or progressive nature, with alteration of multiple higher cortical functions, including memory, thinking, orientation, comprehension, language, ability to learn, perform calculations, and decision making. Awareness is not lost. Deficits in cognitive skills are commonly accompanied, and occasionally preceded, by deterioration of emotional control, social behavior or motivation.^2^ Dementia is characterized by the presence of persistent cognitive impairment that interferes with an individual's ability to perform their professional or social activities. It is independent of the presence of changes in level of awareness and is caused by a disease that affects the central nervous system. Dementia is an acquired disease as the impairment term makes clear. This can be caused by many diseases and while often having slow, progressive and irreversible evolution, it can also be acute or subacute, being reversible by treating the specific underlying disease, when such treatment is available and provided at the right time.^3-5^

Currently, the diagnosis is obtained by using biomarkers and neurogenetics. These findings allow patients to be identified, years or even decades, before reaching the clinical diagnosis of dementia (presymptomatic disease of Alzheimer).^6^ This advance is important because until a few years ago, accurate diagnosis and differentiation of subtypes was possible only through an exam of pathological anatomy.^7,8^

Currently, the DSM-5 defines dementia as a progressive and chronic disease of the central nervous system that affects higher cognitive functions (thinking, language, and memory).^9^

In 2008, the World Health Organization (WHO) published guidelines to expand the treatment of dementia in low- and middle-income countries.^10^ In 2012, it urged governments to consider dementia a public health priority.^11^ In September 2014, through the World Alzheimer Report it was recommended that dementia be integrated into public health programs with other non-communicable chronic diseases.^12^

**Dementia worldwide and in Latin America**. The risk of dementia in the developing world is set to increase quickly over the next 50 years due to the growth in the elderly population (>60 years), unlike other age groups.^11^ Since 1999, a group of experts from Alzheimer's Disease International (ADI) have created a common agenda to counteract the imbalance that exists in research in the field of dementia. Only 10% of population-based research in dementia was performed in low- and middle-income regions, home to 66% of affected individuals, and the group was therefore called the 10/66 group.^13^ The 10/66 Dementia Research Group is a network of researchers around the world, pooling efforts to promote research on dementia in low- and middle- income countries. The number of people with dementia globally in 2013 was estimated at 44 million, expected to reach 76 million in 2030 and 135 million by 2050. The new data represent a 17% increase in global estimates, compared with previous published data in the World Alzheimer's Report which estimated the number of people living with dementia at 35 million in 2010, 66 million in 2030 and 115 million by the year 2050.^10^

These figures reveal a global problem with an impact that affects developing countries whose limited resources and time complicate the development of comprehensive systems of social protection, health and social care. Progress is expected in the treatment of dementia that could mitigate the impact of the next epidemic, but concerted effort is essential to close the gap between diagnosis and treatment. The high-income countries belonging to the G8, formed by the world's most industrialized nations (Germany, Italy, Canada, Japan, USA, UK, France and Russia) have suffered the burden of dementia epidemic.^11^ In the coming decades, most dementia patients will be located in low- and middle-income countries, where 71% of dementia patients reside. The problem is particularly acute in low- to middle-income (LMI) countries, since dementia is contributing to increased disability in the elderly.^14^

The 2010 World Alzheimer Report estimated the annual global cost of dementia at US$ 604 billion. These costs included three components: direct medical care, social care and the indirect costs of informal care provided by unpaid family caregivers.

Dementia has a huge impact on the socio-economic conditions in the world. The cost of $ 604 billion represents 1.0% of all gross domestic product (GDP) collected worldwide. If dementia care were a country, it would be the 18^th^ largest economy, in the same position as Turkey and Indonesia. If It was a company it would be the largest in the world with annual incomes exceeding Wal-Mart ($ 414 billion) and Exxon Mobil (US$ 311 billion)^11^.

The 2013 World Alzheimer Report noted that only 13 countries are currently prepared for the epidemic of dementia, implementing a national plan to tackle the disease.

A collaborative global action plan for governments, industry and non-profit organizations is suggested. It also notes that research must be a global priority toward improving the quality and coverage of care, finding treatments that alter the course of disease and identifying more options for prevention. Policy, the development of health systems and social care services should also be prioritized.^10,15^ By the middle of the 21^st^ century, 78% of the world's elderly population will reside in low- to middle-income countries, with the concomitant expected increase in cases of dementia.^16^

To comprehend the magnitude of the dementia problem the figures must be analyzed. These show that Latin America and the Caribbean (LAC) have the highest prevalence in the world (8.5% in >60 years, whereas in the United States the rate is 6.4%,^10,17^ although most estimates of the prevalence of dementia in people over 60 lie between 5% and 7%.^10^ The variation in the actual prevalence of dementia is influenced in sub-Saharan Africa (2.1%) by the selective mortality of persons under 60 years.^18^ Despite the low prevalences determined in Africa, India and Bangladesh, elderly populations face additional risk factors for dementia such as those related to HIV and traumatic brain injury. These pathologies predict an increase in patients with dementia.^19^

In 2005, Ferri suggested that the prevalence of dementia in developing countries was lower than in developed countries.^20^ However, eight prevalence studies have been conducted in six different Latin American countries, finding a prevalence of 7.1% in the elderly (>65) reflecting the rates of developed countries. The prevalence in relatively young old (65-69 years) was elevated in the Latin American findings. Regarding the risk factor of illiteracy, the rate was 9,3% in the elderly population and the prevalence of dementia in illiterates was twice as high as in literates.^21^

In Latin America, Alzheimer's disease is the leading cause of dementia. Rates vary from 49% in Maracaibo, Venezuela, to 84.5% in Concepción, Chile. The second most common kind of dementia is the vascular type, whose prevalence ranges from 8.7% in Lima, Peru, to 26.5% in Maracaibo, Venezuela.^21^

There are substantial differences in the results of Latin American studies on the prevalence of dementia where rates range from between 2% in a study conducted in Brazil^22^ to 13% in a study of Venezuela.^23^

In Colombia, dementia was reported in epidemiological studies in 1987 by the Neurosciences group of the University of Antioquia, where presenile dementia closely resembling Alzheimer's disease was found in the population under 50 years old. The existence of a DNA mutation was identified explaining the condition, with an autosomal dominant transmission mode which implies a 50% risk in all children of those affected.^24^

In Bogota, the Health Ministry ran a pilot study on the prevalence of dementias in the community published in 2000.^25^ Although the results show an ostensible high prevalence "it is not valid, since the sample was not reliable and therefore does not reflect the Bogota population". Nevertheless, from the standpoint of validation of a community outreach strategy as well as use and adaptation of diagnostic techniques, this study represents an important precedent for the development of future projects.^26^

Subsequently, a study for Pradilla et al.^27^ between 1995 and 1996 was performed, in which a sample of 8,910 people divided into five geographic areas was examined: central, southwestern, northwestern, eastern and the Colombian Caribbean coast. A neuropsychological test was applied. The Hachinski,^28^ Yesavage,^29^ and Blessed^30^ scales, along with the NINCDS-ADRDA diagnostic criteria for Alzheimer's disease,^7^ the NINDS-AIREN criteria for vascular dementia^31^ and DSM-IV for all types of dementia were applied. The prevalence of eight neurological disorders was determined in the general population, including the overall prevalence of dementia: 13.1 per thousand (95%CI: 8.5 to 19.3).

In the city of Neiva, Huila, Colombia, in the 2003-2005 period, Gooding et al.^26^ conducted a socio-demographic and clinical investigation of behavior in people aged 60 or older to establish the prevalence of dementia and associated risk factors. The quantitative cross-sectional descriptive study and sample design was implemented in two consecutive phases (screening and diagnostic testing) in a random sample of 643 people. In the first phase (screening), individuals with cognitive impairment were identified through a demographic survey, the mini-mental state examination extended version of Ashford (MMSE/50) which contains all the original Folstein plus an extension of 20 items on cognition, a scale assessing the performance of instrumental activities of daily living (IADL) (Lawton) and the Geriatric Depression Scale (Yesavage). All patients with probable cognitive impairment impacting IADL continued to the second stage, where diagnostic testing was performed. This entailed a multidisciplinary clinical examination consisting of a neuropsychological review with the implementation of a large protocol (Antioquia Neuroscience Group, U. of A.), the CERAD (Consortium to Establish a Registry for Alzheimer's Disease) battery and additional tests such as the Wisconsin, Rey-Osterrieth complex figure, and scale of subjective memory complaints. The final diagnosis of dementia was reached by the interdisciplinary team applying the algorithm and diagnostic criteria agreed: GDS (Global Deterioration Scale), DSM IV (Diagnostic and Statistical Manual of Mental Disorders of the American Psychiatric Association), NINCDS-ADRA (diagnostic criteria of the National Institute of Neurological Disorders and Alzheimer's Disease and Related Disorders) and NINDS AIREN (Diagnostic Criteria for Vascular Dementia: National Institute of Neurological Disorders and Stroke and Association Internationale pour la Recherche et l' Enseignement in Neurosciences). Subjects having physical (motor, sensory, neurological or other) or mental (mental retardation, psychiatric illness, major depression) illnesses diagnosed or evident which limited their performances on the tests were excluded. In the first stage involving the MMSE, the results indicated that if the original MMSE had been used as the screening test, 31% of individuals who continued to the second phase with some level of cognitive impairment would have been classified as normal with the version. This indicated that the extended version MMSE/50 eliminated 31% of the false negatives, showed greater sensitivity than the original version and was established as a good screening tool. At this stage, 219 subjects (34%) were classified with some degree of cognitive impairment. In the second phase of this selected group, only 170 individuals remained (77.7%) after elimination of 22% based on exclusion criteria and 152 people were classified as having probable dementia because at the time the only definitive diagnosis was reached by neuropathological examination. The number (152) with probable dementia corresponded to 23.6%. This represents the highest prevalence detected in the country thus far. This prevalence was represented by 59.9% dementia of degenerative dementia and 40% mixed, vascular and other unspecified types.

The risk factors identified included illiteracy, low education level, low socioeconomic status (21.9%), history of high blood pressure (92%), diabetes mellitus (86.8%), dyslipidemias (82.2%), depression (32.9%), cardiovascular disease (13.5%), and cerebrovascular disease (7.3%). A total of 81% of those with dementia had low levels of schooling: 44% were illiterate and 37% had fewer than three years of schooling, an alarmingly high figure. The study shows that one of the most important risk factors is low education which is why some authors have proposed that people with more schooling are resistant to the effects of dementing processes as a result of a greater cognitive reserve^32^ which represents a modulator of neurodegenerative processes and clinical manifestations of cognitive decline and dementia. This is associated with the ability to optimize the execution of tasks through the recruitment of neural networks and use of alternative cognitive strategies mediated by formal education processes. Reading level has been shown as a good measure of cognitive reserve and a reliable predictor of executive and cognitive functioning in the aging process.^33^

High school education has been shown to be a protective factor. Thus, in an assessment of dementia in a highly-educated population in Tianjin, China, the prevalence was lower in the elderly with high school education, suggesting an association between lower cognitive activity and presence of dementia.^34^ Regarding illiteracy as a risk factor, in Latin America a rate of 9.3% is reported among older adults^35,36^ and the prevalence of dementia in illiterates is twice that of literates.^21^ However, studies that included more than 15,000 people without dementia in six Latin American countries, China and India, did not check the association between cognitive impairment and age or education but confirmed the presence of neuropsychiatric disorders among people with cognitive impairment who progressed to dementia.^37^

As regard to clinical risk factors, hypertension was considered a major risk factor for the incidence of dementia and it has also been suggested that its therapeutic control can reduce the risk of developing dementia in old age by 3-20%.^35,36,38^ The World Alzheimer Report 2014 reveals that diabetes can increase the risk of dementia by 50%.^12^

On the other hand, nutritional deficit plays an important role in the process of brain aging and resultant cognitive impairment. It has been reported that the Mediterranean diet, characterized by high consumption of vegetables, fruits, nuts and grains, is linked to a reduced risk of Alzheimer's disease and lower mortality of patients with the disease.^39-41^

Taking into account the high prevalence of dementia in Neiva (23.6%), we considered it appropriate to produce a short synopsis in a bid to analyze the demographics and cultural issues that may be affecting these numbers.

## HUILA STATE OVERVIEW

The city of Neiva is the capital of the Huila Department. Huila was founded in 1905 and is located in the southern Andean region, consisting of 37 municipalities with an area of 19,890 km^2^ representing only 1.8% of the total area of the country. It has an average temperature of 24ºC with a warmer central department, especially in the valley of Neiva which averages 28ºC, extending to the north end throughout the semiarid region of Yararaca (Tatacoa). It had a population of 1,126,316 inhabitants in 2013, 59.9% of which is located in the urban municipalities and 40.1% in rural areas. The average population density in the department is 50.86 inhabitants/km^2^. In 2012 Neiva had a population of 337,848 inhabitants. The composition of the department's population is multiracial: 97.78% Mestizos and Whites, 1.17% Blacks and Afro-Colombians and 1.05% Amerindians or Indians.

By gender, the department's population in 2013 was 50.1% male and 49.9% female. The average population density of the capital, Neiva, is 231 km^2^.

The population of the department of Huila is expansive in nature, showing a high concentration of young active population and labor, plus a decline in the adult and elderly population, although projections for 2020 show a pattern of decline in young people and rise in the number of adults.^42^ The crude death rate fell from 20.6 per thousand in 2005 to 18.2 per thousand in 2011, while the birth rate remains stable at 4.5 per 1,000 inhabitants in 2005 versus 4.3 per 1,000 inhabitants in 2011.^43^

The age groups from youth to seniors, by contrast, show a percentage increase, sustained in adulthood and in the elderly. Adults will present an increase of 3.8% and greater than 2.5% increase, while the young show a minimum increase of 0.1% in the 2005-2020 period (Table 1). These changes can be explained by the behavior of fertility in the department. The total fertility rate of 83.3 births per 1000 women aged 15-4t9 years in 2005 fell to 71.5 births per 1,000 women in the same age group in 2013.

**Table 1:** Proportion of the population life cycle Huila department 2005, 2013 and 2020.

Life cycle	2005			2013			2020	
	Absolute number	Relative frequency		Absolute number	Relative frequency		Absolute number	Relative frequency
Early childhood (0-5 years)	138,434	13.7%		135,759	12.1%		139,298	11.4%
Infancy (6-11 years)	143,127	14.2%		135,027	12.0%		135,194	11.0%
Adolescent (12-18 years)	149,842	14.8%		160,554	14.3%		156,177	12.7%
Young (14-26 years)	238,338	23.6%		280,875	24.9%		290,038	23.7%
Adult (27-59 years)	358,426	35.4%		420,700	37.4%		480,492	39.2%
Elderly (≥60 years)	86,732	8.6%		108,382	9.6%		135,734	11.1%
Total Huila*	1,011,405	100		1,126,316	100		1,225,343	100

**Figure 1:**
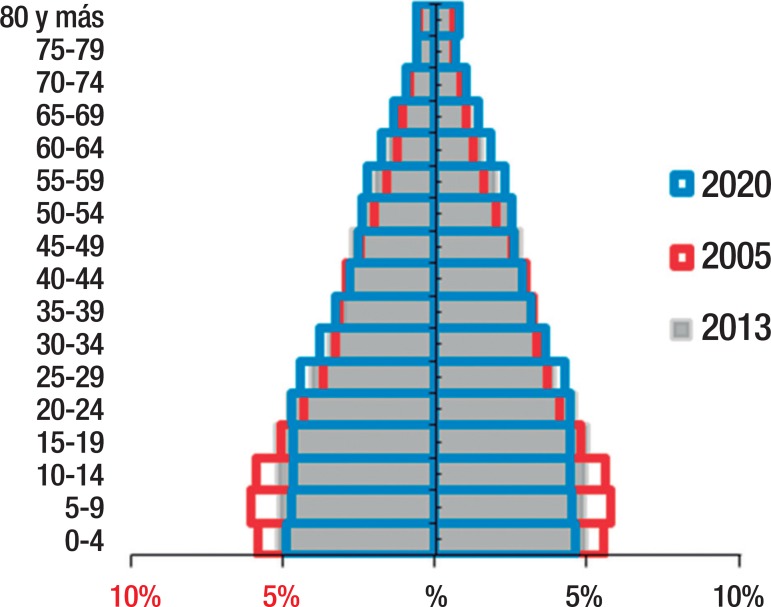
Population pyramid, Department of Huila, 2005, 2013, 2020

Life expectancy at birth for the 2005-2010 period was 74 years and is set to rise by two years to 76 for the 2015-2020 period according to the Health Situation Analysis (ASIS - Analisis de la Situación de Salud - HUILA, 2011). This increase in life expectancy at birth reflects increased survival and therefore greater representation in the population pyramid of adult age groups and older people (Figure 1). The proportion of the elderly population by 2020 will be the same as that of infants and children. Other possible explanations for this dynamic is the decrease in the specific mortality rate associated with violence (from 75 per 100,000 population in 2005 to about 50 per 100,000 inhabitants in 2011) and increased survival associated with non-communicable diseases with high prevalence, thus allowing younger adults and elderly to reach the cycle. Therefore, Huila is undergoing a slow demographic transition from a clearly wide expansive population pyramid to a narrow-based stationary population pyramid.

**Conclusions**. The high prevalence of dementia in Latin America and the Caribbean (8.5%) could be associated with the confluence of genetic or environmental risk factors.^41^ For example, the increased mobility of individuals and families has expanded genetic isolates, and early-onset Alzheimer's disease (Medellin, Colombia)^44^ and Huntington's disease in Maracaibo, Venezuela.^45^

The fact that the expression of the disease occurs after reproductive age may also contribute to its spread. Finally, in consanguineous marriages, relatively common in some poor areas, dementia in one or both parents increases the risk of having children who will also be affected in the future.^46^

Local governments are unlikely to change their current focus from malnutrition and communicable diseases to an "incurable" disease and dementia. In addition, the prevalence of many preventable chronic diseases such as diabetes and cardiovascular increases as the low- to middle-income countries adopt westernized life styles, further increasing pressure on limited health resources.^19^

Although the dental status of our study population was not included, the only data on the prevalence of edentulism in Colombia from a study conducted in1998 revealed 25% edentulism in both jaws and 7% in the lower jaw only.^47^ This is important to consider because an association between edentulism and cognitive status has been found in industrialized countries as well as in some rural populations in developing countries.^17,46-50^

While the figure of 8.5% prevalence in Latin America and the Caribbean is considered high, finding a figure almost triple (23.6%) this in the city of Neiva^26^ can be explained by several factors. These are the inclusion of four geriatric homes and also the inverse population pyramid, in addition to the presence of risk factors such as low education and poverty. Low education implies low cognitive reserve. The 37% with less than 3 years of schooling coupled with an illiteracy rate of up to 44% gives a total low schooling rate of 81%. In addition, it was found that 82.9% had difficult living conditions, belonging to low socio-economic strata associated with chronic diseases such as diabetes and high blood pressure, heart disease, known to have a marked influence on vascular function and anatomy, and hence on brain irrigation and oxygenation. This high prevalence may be alarming but was reached based on a rigorously designed study where participants underwent proper and perhaps the most comprehensive screening at the time for early diagnosis. Finally, we believe there could be other unknown risk factors and etiologies influencing this high prevalence.
